# *N*-(2-Chloro-4-methyl­phen­yl)succinamic acid

**DOI:** 10.1107/S1600536812005648

**Published:** 2012-02-17

**Authors:** U. Chaithanya, Sabine Foro, B. Thimme Gowda

**Affiliations:** a Department of Chemistry, Mangalore University, Mangalagangotri 574 199, Mangalore, India; b Institute of Materials Science, Darmstadt University of Technology, Petersenstrasse 23, D-64287 Darmstadt, Germany

## Abstract

In the title compound, C_11_H_12_ClNO_3_, the N—C=O fragment is twisted from the plane of the attached benzene ring by 48.39 (12)°. The carb­oxy­lic acid group is involved in O—H⋯O hydrogen bonding, which links pairs of mol­ecules into centrosymmetric dimers. N—H⋯O hydrogen bonds link these dimers, related by translation along the *a* axis, into ribbons.

## Related literature

For the crystal structures of related compounds studied by our group, see: Gowda *et al.* (2012 ▶) and references therein.
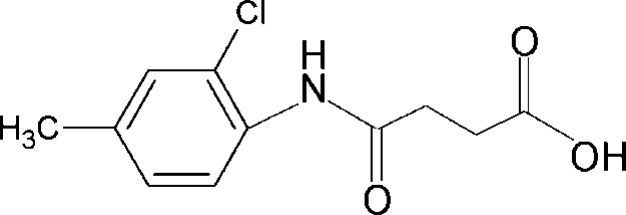


## Experimental

### 

#### Crystal data

C_11_H_12_ClNO_3_
*M* *_r_* = 241.67
Triclinic, 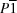

*a* = 4.8097 (8) Å
*b* = 7.3909 (9) Å
*c* = 16.147 (2) Å
α = 85.15 (1)°
β = 85.86 (1)°
γ = 89.57 (1)°
*V* = 570.45 (14) Å^3^
*Z* = 2
Mo *K*α radiation
μ = 0.33 mm^−1^
*T* = 293 K
0.40 × 0.18 × 0.09 mm

#### Data collection

Oxford Xcalibur diffractometer with Sapphire CCD detector
Absorption correction: multi-scan (*CrysAlis RED*; Oxford Diffraction, 2009 ▶) *T*_min_ = 0.881, *T*_max_ = 0.971
3625 measured reflections
2284 independent reflections
1883 reflections with *I* > 2σ(*I*)
*R*_int_ = 0.011

#### Refinement

*R*[*F* ^2^ > 2σ(*F* ^2^)] = 0.053
*wR*(*F* ^2^) = 0.120
*S* = 1.11
2284 reflections
152 parameters
2 restraints
H atoms treated by a mixture of independent and constrained refinement
Δρ_max_ = 0.28 e Å^−3^
Δρ_min_ = −0.25 e Å^−3^

### 

Data collection: *CrysAlis CCD* (Oxford Diffraction, 2009 ▶); cell refinement: *CrysAlis RED* (Oxford Diffraction, 2009 ▶); data reduction: *CrysAlis RED*; program(s) used to solve structure: *SHELXS97* (Sheldrick, 2008 ▶); program(s) used to refine structure: *SHELXL97* (Sheldrick, 2008 ▶); molecular graphics: *PLATON* (Spek, 2009 ▶); software used to prepare material for publication: *SHELXL97*.

## Supplementary Material

Crystal structure: contains datablock(s) I, global. DOI: 10.1107/S1600536812005648/cv5244sup1.cif

Structure factors: contains datablock(s) I. DOI: 10.1107/S1600536812005648/cv5244Isup2.hkl

Supplementary material file. DOI: 10.1107/S1600536812005648/cv5244Isup3.cml

Additional supplementary materials:  crystallographic information; 3D view; checkCIF report

## Figures and Tables

**Table 1:** Hydrogen-bond geometry (Å, °)

*D*—H⋯*A*	*D*—H	H⋯*A*	*D*⋯*A*	*D*—H⋯*A*
N1—H1N⋯O1^i^	0.84 (2)	2.16 (2)	2.973 (3)	163 (3)
O3—H3*O*⋯O2^ii^	0.83 (2)	1.85 (2)	2.674 (3)	172 (4)

Symmetry codes: (i) 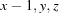; (ii) 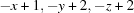.

**Fig. 1.**
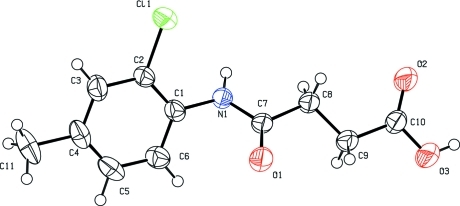
The molecular structure of (I) showing the atom labelling scheme and 50% probability displacement ellipsoids.

**Fig. 2.**
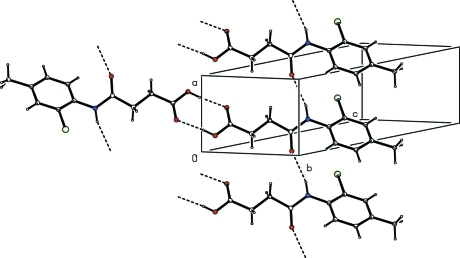
A portion of the crystal packing with hydrogen bonds shown as dashed lines.

## supplementary crystallographic information

### Comment

Recently, we reported the crystal structure of *N*-(2-chloro-5-methylphenyl)succinamic acid (Gowda *et al.*, 2012). We report here the crystal structure of very closely related *N*-(2-chloro-4-methylphenyl)succinamic acid, (I).

In (I) (Fig. 1), all bond lengths and angles are normal and correspond to those observed in related compounds (Gowda *et al.*, 2012, and references therein). The conformations of the amide oxygen and the carboxyl oxygen of the acid segment are *anti* to each other and both are *anti* to the H atoms on the adjacent –CH_2_ group.. The C═O and O—H bonds of the acid group are in *syn* position to each other. The dihedral angle between the benzene ring and the amide group is 48.39 (12)°.

The packing of molecules in the crystal through intermolecular O—H···O and N—H···O hydrogen bonds (Table 1) is shown in Fig. 2.

### Experimental

The solution of succinic anhydride (0.01 mole) in toluene (25 ml) was treated dropwise with the solution of 2-chloro-4-methylaniline (0.01 mole) also in toluene (20 ml) with constant stirring. The resulting mixture was stirred for about one hour and set aside for an additional hour at room temperature for completion of the reaction. The mixture was then treated with dilute hydrochloric acid to remove the unreacted 2-chloro-4-methylaniline. The resultant title compound was filtered under suction and washed thoroughly with water to remove the unreacted succinic anhydride and succinic acid. It was recrystallized to constant melting point from ethanol. The purity of the compound was checked and characterized by its infrared and NMR spectra. Rod like colourless single crystals used in X-ray diffraction studies were grown in ethanol solution by slow evaporation at room temperature.

### Refinement

The H atoms of the NH and OH groups were located in a difference map and restrained to the distances N—H = 0.86 (2) Å and O—H = 0.82 (2) Å, respectively. The other H atoms were positioned with idealized geometry using a riding model with the aromatic C—H = 0.93 Å and methylene C—H = 0.97 Å. All H atoms were refined with isotropic displacement parameters set to 1.2–1.5*U*_eq_ of the parent atom.

### Crystal data

**Table:**

C_11_H_12_ClNO_3_	*Z* = 2
*M**_r_* = 241.67	*F*(000) = 252
Triclinic, *P*1	*D*_x_ = 1.407 Mg m^−^^3^
Hall symbol: -P 1	Mo *K*α radiation, λ = 0.71073 Å
*a* = 4.8097 (8) Å	Cell parameters from 2016 reflections
*b* = 7.3909 (9) Å	θ = 2.5–27.9°
*c* = 16.147 (2) Å	µ = 0.33 mm^−^^1^
α = 85.15 (1)°	*T* = 293 K
β = 85.86 (1)°	Rod, colourless
γ = 89.57 (1)°	0.40 × 0.18 × 0.09 mm
*V* = 570.45 (14) Å^3^	

### Data collection

**Table:**

Oxford Xcalibur diffractometer with Sapphire CCD detector	2284 independent reflections
Radiation source: fine-focus sealed tube	1883 reflections with *I* > 2σ(*I*)
Graphite monochromator	*R*_int_ = 0.011
Rotation method data acquisition using ω and φ scans	θ_max_ = 26.4°, θ_min_ = 2.5°
Absorption correction: multi-scan (*CrysAlis RED*; Oxford Diffraction, 2009)	*h* = −5→5
*T*_min_ = 0.881, *T*_max_ = 0.971	*k* = −9→9
3625 measured reflections	*l* = −17→20

### Refinement

**Table:**

Refinement on *F*^2^	Primary atom site location: structure-invariant direct methods
Least-squares matrix: full	Secondary atom site location: difference Fourier map
*R*[*F*^2^ > 2σ(*F*^2^)] = 0.053	Hydrogen site location: inferred from neighbouring sites
*wR*(*F*^2^) = 0.120	H atoms treated by a mixture of independent and constrained refinement
*S* = 1.11	*w* = 1/[σ^2^(*F*_o_^2^) + (0.0348*P*)^2^ + 0.5262*P*] where *P* = (*F*_o_^2^ + 2*F*_c_^2^)/3
2284 reflections	(Δ/σ)_max_ = 0.001
152 parameters	Δρ_max_ = 0.28 e Å^−^^3^
2 restraints	Δρ_min_ = −0.25 e Å^−^^3^

### Special details

**Table:**

Experimental. CrysAlis RED (Oxford Diffraction, 2009) Empirical absorption correction using spherical harmonics, implemented in SCALE3 ABSPACK scaling algorithm.
Geometry. All e.s.d.'s (except the e.s.d. in the dihedral angle between two l.s. planes) are estimated using the full covariance matrix. The cell e.s.d.'s are taken into account individually in the estimation of e.s.d.'s in distances, angles and torsion angles; correlations between e.s.d.'s in cell parameters are only used when they are defined by crystal symmetry. An approximate (isotropic) treatment of cell e.s.d.'s is used for estimating e.s.d.'s involving l.s. planes.
Refinement. Refinement of *F*^2^ against ALL reflections. The weighted *R*-factor *wR* and goodness of fit *S* are based on *F*^2^, conventional *R*-factors *R* are based on *F*, with *F* set to zero for negative *F*^2^. The threshold expression of *F*^2^ > σ(*F*^2^) is used only for calculating *R*-factors(gt) *etc*. and is not relevant to the choice of reflections for refinement. *R*-factors based on *F*^2^ are statistically about twice as large as those based on *F*, and *R*-factors based on ALL data will be even larger.

### Fractional atomic coordinates and isotropic or equivalent isotropic displacement parameters (Å^2^)

**Table:**

	*x*	*y*	*z*	*U*_iso_*/*U*_eq_	
C1	0.7464 (5)	0.1423 (3)	0.72556 (16)	0.0372 (5)	
C2	0.6617 (5)	0.1119 (3)	0.64792 (16)	0.0397 (6)	
C3	0.7600 (6)	−0.0346 (3)	0.60606 (18)	0.0479 (6)	
H3	0.6972	−0.0528	0.5543	0.057*	
C4	0.9510 (6)	−0.1542 (4)	0.64083 (19)	0.0516 (7)	
C5	1.0408 (6)	−0.1206 (4)	0.7175 (2)	0.0544 (7)	
H5	1.1728	−0.1976	0.7412	0.065*	
C6	0.9412 (5)	0.0232 (4)	0.76017 (18)	0.0465 (6)	
H6	1.0040	0.0408	0.8120	0.056*	
C7	0.7910 (5)	0.4059 (3)	0.80500 (15)	0.0386 (6)	
C8	0.6293 (5)	0.5544 (4)	0.84608 (17)	0.0431 (6)	
H8A	0.4480	0.5081	0.8679	0.052*	
H8B	0.5999	0.6544	0.8047	0.052*	
C9	0.7800 (5)	0.6234 (4)	0.91616 (17)	0.0449 (6)	
H9A	0.7896	0.5262	0.9602	0.054*	
H9B	0.9696	0.6544	0.8955	0.054*	
C10	0.6475 (5)	0.7848 (4)	0.95222 (16)	0.0441 (6)	
C11	1.0540 (8)	−0.3163 (4)	0.5961 (2)	0.0790 (11)	
H11A	1.0422	−0.2903	0.5371	0.095*	
H11B	0.9409	−0.4201	0.6151	0.095*	
H11C	1.2442	−0.3414	0.6075	0.095*	
N1	0.6355 (4)	0.2886 (3)	0.76914 (14)	0.0404 (5)	
H1N	0.463 (4)	0.306 (4)	0.7706 (17)	0.049*	
O1	1.0450 (3)	0.3984 (3)	0.80286 (14)	0.0594 (6)	
O2	0.4442 (4)	0.8624 (3)	0.92593 (15)	0.0741 (7)	
O3	0.7748 (6)	0.8378 (4)	1.01368 (17)	0.0888 (9)	
H3O	0.696 (8)	0.924 (4)	1.035 (2)	0.107*	
Cl1	0.42759 (15)	0.26087 (10)	0.60111 (5)	0.0573 (2)	

### Atomic displacement parameters (Å^2^)

**Table:**

	*U*^11^	*U*^22^	*U*^33^	*U*^12^	*U*^13^	*U*^23^
C1	0.0262 (11)	0.0349 (12)	0.0506 (14)	0.0017 (9)	0.0016 (10)	−0.0079 (10)
C2	0.0289 (12)	0.0395 (13)	0.0520 (15)	0.0062 (10)	−0.0033 (10)	−0.0110 (11)
C3	0.0484 (15)	0.0411 (14)	0.0554 (16)	0.0046 (12)	0.0006 (12)	−0.0164 (12)
C4	0.0498 (16)	0.0356 (13)	0.0685 (19)	0.0076 (11)	0.0104 (14)	−0.0118 (12)
C5	0.0447 (15)	0.0441 (15)	0.073 (2)	0.0164 (12)	−0.0009 (14)	0.0012 (14)
C6	0.0374 (14)	0.0482 (15)	0.0540 (16)	0.0057 (11)	−0.0033 (11)	−0.0056 (12)
C7	0.0255 (12)	0.0447 (13)	0.0474 (14)	0.0032 (10)	−0.0044 (10)	−0.0137 (11)
C8	0.0266 (12)	0.0492 (14)	0.0570 (16)	0.0044 (10)	−0.0076 (11)	−0.0217 (12)
C9	0.0332 (13)	0.0532 (15)	0.0516 (15)	0.0059 (11)	−0.0090 (11)	−0.0192 (12)
C10	0.0353 (13)	0.0533 (15)	0.0463 (14)	−0.0003 (11)	−0.0036 (11)	−0.0192 (12)
C11	0.094 (3)	0.0468 (17)	0.095 (3)	0.0276 (17)	0.015 (2)	−0.0168 (17)
N1	0.0214 (9)	0.0463 (12)	0.0568 (13)	0.0056 (8)	−0.0042 (9)	−0.0223 (10)
O1	0.0219 (9)	0.0689 (13)	0.0934 (16)	0.0049 (8)	−0.0080 (9)	−0.0395 (12)
O2	0.0578 (13)	0.0868 (16)	0.0892 (16)	0.0282 (12)	−0.0307 (12)	−0.0551 (13)
O3	0.0870 (18)	0.0996 (19)	0.0954 (19)	0.0402 (14)	−0.0513 (15)	−0.0655 (16)
Cl1	0.0547 (4)	0.0582 (4)	0.0627 (5)	0.0234 (3)	−0.0177 (3)	−0.0183 (3)

### Geometric parameters (Å, º)

**Table:**

C1—C2	1.382 (3)	C7—C8	1.512 (3)
C1—C6	1.395 (3)	C8—C9	1.512 (3)
C1—N1	1.420 (3)	C8—H8A	0.9700
C2—C3	1.387 (3)	C8—H8B	0.9700
C2—Cl1	1.736 (2)	C9—C10	1.490 (3)
C3—C4	1.385 (4)	C9—H9A	0.9700
C3—H3	0.9300	C9—H9B	0.9700
C4—C5	1.383 (4)	C10—O2	1.215 (3)
C4—C11	1.513 (4)	C10—O3	1.292 (3)
C5—C6	1.379 (4)	C11—H11A	0.9600
C5—H5	0.9300	C11—H11B	0.9600
C6—H6	0.9300	C11—H11C	0.9600
C7—O1	1.221 (3)	N1—H1N	0.839 (17)
C7—N1	1.343 (3)	O3—H3O	0.827 (19)
			
C2—C1—C6	117.9 (2)	C9—C8—H8A	109.2
C2—C1—N1	121.0 (2)	C7—C8—H8B	109.2
C6—C1—N1	121.1 (2)	C9—C8—H8B	109.2
C1—C2—C3	121.6 (2)	H8A—C8—H8B	107.9
C1—C2—Cl1	119.42 (18)	C10—C9—C8	114.3 (2)
C3—C2—Cl1	118.9 (2)	C10—C9—H9A	108.7
C4—C3—C2	120.5 (3)	C8—C9—H9A	108.7
C4—C3—H3	119.8	C10—C9—H9B	108.7
C2—C3—H3	119.8	C8—C9—H9B	108.7
C5—C4—C3	117.7 (2)	H9A—C9—H9B	107.6
C5—C4—C11	121.7 (3)	O2—C10—O3	122.8 (2)
C3—C4—C11	120.6 (3)	O2—C10—C9	124.0 (2)
C6—C5—C4	122.2 (3)	O3—C10—C9	113.2 (2)
C6—C5—H5	118.9	C4—C11—H11A	109.5
C4—C5—H5	118.9	C4—C11—H11B	109.5
C5—C6—C1	120.0 (3)	H11A—C11—H11B	109.5
C5—C6—H6	120.0	C4—C11—H11C	109.5
C1—C6—H6	120.0	H11A—C11—H11C	109.5
O1—C7—N1	123.2 (2)	H11B—C11—H11C	109.5
O1—C7—C8	121.7 (2)	C7—N1—C1	124.10 (19)
N1—C7—C8	115.08 (19)	C7—N1—H1N	117.9 (19)
C7—C8—C9	111.9 (2)	C1—N1—H1N	117.9 (19)
C7—C8—H8A	109.2	C10—O3—H3O	113 (3)
			
C6—C1—C2—C3	1.7 (4)	C2—C1—C6—C5	−0.8 (4)
N1—C1—C2—C3	−177.4 (2)	N1—C1—C6—C5	178.4 (2)
C6—C1—C2—Cl1	−178.02 (19)	O1—C7—C8—C9	−28.9 (4)
N1—C1—C2—Cl1	2.8 (3)	N1—C7—C8—C9	153.3 (2)
C1—C2—C3—C4	−1.0 (4)	C7—C8—C9—C10	173.0 (2)
Cl1—C2—C3—C4	178.8 (2)	C8—C9—C10—O2	−3.6 (4)
C2—C3—C4—C5	−0.7 (4)	C8—C9—C10—O3	177.9 (3)
C2—C3—C4—C11	178.7 (3)	O1—C7—N1—C1	0.6 (4)
C3—C4—C5—C6	1.7 (4)	C8—C7—N1—C1	178.4 (2)
C11—C4—C5—C6	−177.8 (3)	C2—C1—N1—C7	−132.0 (3)
C4—C5—C6—C1	−1.0 (4)	C6—C1—N1—C7	48.9 (4)

### Hydrogen-bond geometry (Å, º)

**Table:**

*D*—H···*A*	*D*—H	H···*A*	*D*···*A*	*D*—H···*A*
N1—H1*N*···O1^i^	0.84 (2)	2.16 (2)	2.973 (3)	163 (3)
O3—H3*O*···O2^ii^	0.83 (2)	1.85 (2)	2.674 (3)	172 (4)

Symmetry codes: (i) *x*−1, *y*, *z*; (ii) −*x*+1, −*y*+2, −*z*+2.
